# Child marriage and health disparities in adulthood: the differential risk of untreated hypertension among young adult women in India

**DOI:** 10.1186/s40885-022-00213-6

**Published:** 2022-10-15

**Authors:** Biplab Kumar Datta, Mohammad Rifat Haider

**Affiliations:** 1 Institute of Public and Preventive Health, Augusta University, Augusta, GA USA; 2 Department of Population Health Sciences, Medical College of Georgia, Augusta University, Augusta, GA USA; 3 Department of Health Policy and Management, College of Public Health, University of Georgia, Athens, GA USA

**Keywords:** Hypertension, Marriage, Women’s health, Healthcare disparities, India

## Abstract

**Background:**

Hypertension is a major risk factor of cardiovascular diseases, which is the leading cause of premature mortality worldwide. While untreated hypertension heightens the risk of mortality and morbidity among hypertensive individuals, access to hypertension care in low-and-middle income countries has ties with various socioeconomic inequalities. Child brides represent a marginalized group of population who experience various socioeconomic disadvantages. This study investigates whether there exists any disparity in receiving treatment for hypertension between child brides at young adult age and their same-age peers who were married as adults.

**Methods:**

We obtained data on 22,140 currently married hypertensive women aged 20 to 34 years from the 2015–16 wave of National Family Health Survey (NFHS-4) of India. We estimated multilevel univariate and multivariable logistic regressions to obtain the odds in favor of not receiving treatment for hypertension. We compared the odds for child brides with those of their peers who were married as adults.

**Results:**

Among the study participants, 72.6% did not receive any treatment for hypertension. While the share was 70.6% among women who were married as adults, it was 4.3 percentage points higher (*P* < 0.001) among the child brides. Results from the multilevel logistic regressions reveal that adjusted odds of having untreated hypertension for child brides were 1.12 times (95% confidence interval, 1.00–1.25) that of those who were married as adults.

**Conclusions:**

Our findings show that hypertensive women who were married as children are at greater risk of not receiving hypertension care at young adult age. Therefore, young women who got married in their childhood should be targeted for regular screening and proper referral and treatment to avoid further detrimental effects of elevated blood pressure.

## Background

Hypertension or elevated blood pressure is the leading cause of cardiovascular diseases and mortality worldwide [1]. Almost one-sixth of the world population (1.28 billion out of total 7.67 billion) are hypertensive, and two-thirds of these populations live in low-and-middle income countries (LMICs) [2]. Alarmingly, almost half of the hypertensive population of the world is unaware about their hypertension [2]. India, a LMIC in South Asia with a large population of 1.3 billion, has a huge burden of noncommunicable diseases (NCDs) [3], and hypertension is one of the greatest risk factors for NCD burden in India [4].

In a systematic review Anchala et al. [5] found that prevalence of hypertension in India was 29.8% and urban population suffered more from hypertension than their rural counterparts. According to the India National Family Health Survey (NFHS-4) conducted in 2015–16, overall hypertension prevalence was 11.3% with 13.8% men aged 15 to 54 years versus 8.8% women aged 15 to 49 years suffering from hypertension [6]. Among the hypertensive population in India less than half (44.7%) were aware of their hypertensive status and less than one (13.3%) in every seven hypertensive individuals were treated [7]. While the rate of treatment is higher among reproductive age women than men, more than 70% women remain untreated for hypertension in India [7].

India is home to 223 million child brides—one-third of the currently living women in the world who were married as children [8]. The practice of child marriage leads to adolescent childbearing, which was found associated with higher risk of hypertension in women at adult age [9]. From 2000 to 2017, ischemic heart disease attributable mortality in Indian women increased more than that in Indian men [10]. As such, Indian women who got married and bore child in their adolescence are at elevated risk of hypertension induced morbidity and mortality. Managing hypertension, on the other hand, is challenging in LMICs like India due to socioeconomic inequalities contributing to inadequate access to care and lack of knowledge [11]. Identifying the population at greater risk of not receiving hypertension care, therefore, has important implications for improving hypertension management in the LMICs.

Child marriage is associated with various socioeconomic disadvantages including lower educational attainment, limited labor force participation and economic opportunities, and lack of voice and agency [12]. Many of these socioeconomic issues are also related to the barriers of hypertension management in the LMICs [11]. Given the increased burden of hypertension among Indian women and the relatively high prevalence of child marriage widening the inequality, it is worthwhile to investigate whether child brides in India are at heightened risk of not receiving hypertension care. Moreover, though the socioeconomic consequences of child marriage are widely studied in literature, there is a dearth of evidence concerning long-term health disparities associated with child marriage [13]. This paper intends to address this gap by examining the disparity in receiving hypertension treatment between child brides at young adult age and their same-age peers who were married as adults.

The specific aim of this paper is to investigate whether child brides at young adulthood (age, 20–34 years) have a differential risk of not receiving hypertension treatment compared to their peers who were married as adults. The findings of this study will inform policies for targeted hypertension prevention and control interventions in low resource settings.

## Methods

### Data

**Fig. 1:**
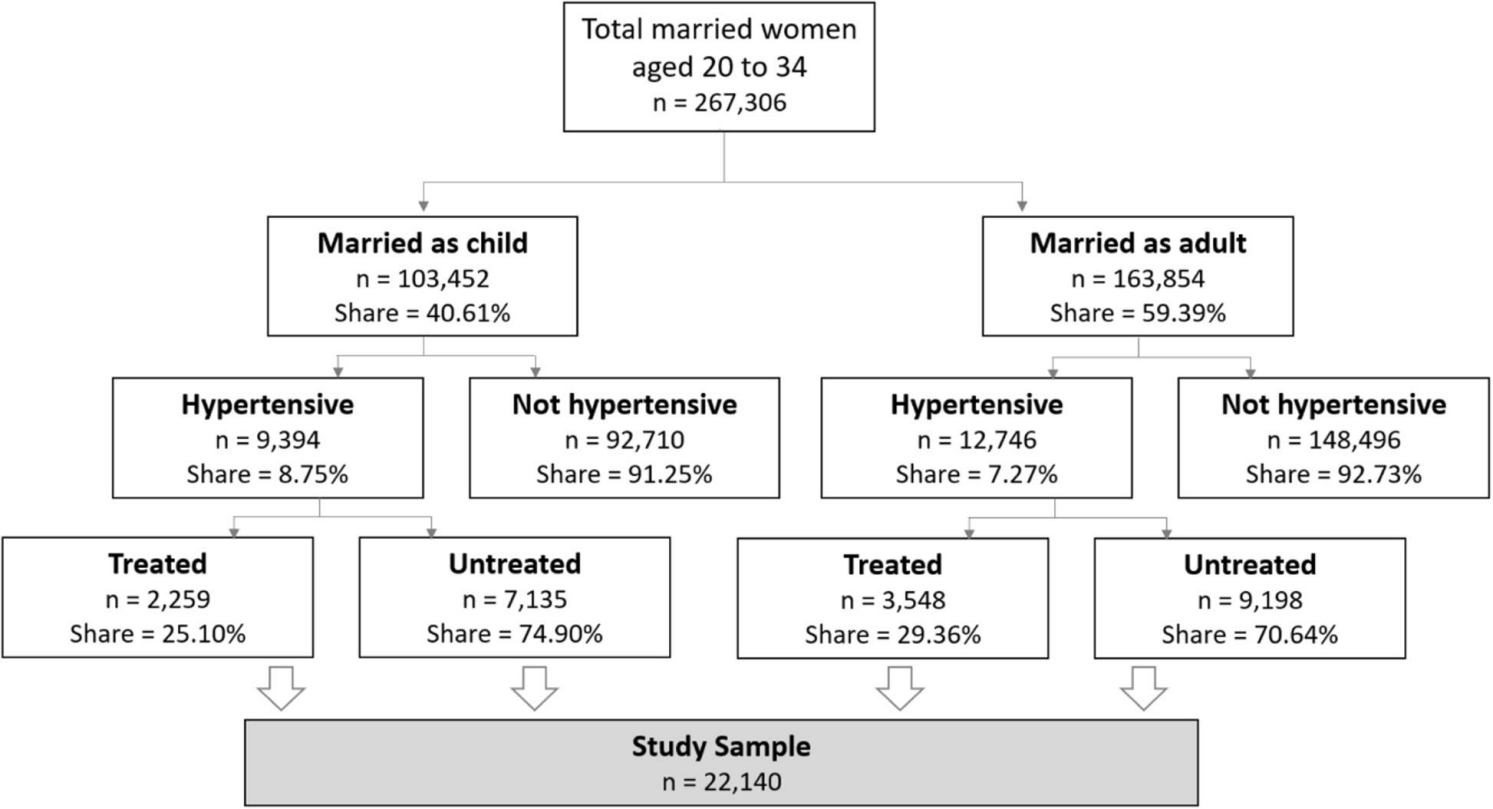
Study sample. Shares were estimated using complex survey weights. The analytical sample includes hypertensive respondents only (*n* = 9,394 + 12,746 = 22,140)

We used data from the NFHS-4, a nationally representative survey that collected health and sociodemographic information of reproductive age women from 640 districts in all the 29 states and seven union territories (total 36) in India using a stratified two-stage sampling framework [14]. Participation in the NFHS-4 was voluntary and consent was obtained prior interview; the survey protocols were reviewed and approved by the Institutional Review Boards of International Institute for Population Sciences and ICF and further reviewed by the US Centers for Disease Control and Prevention [14]. We used anonymized publicly available data for analysis. Our analytical sample contains hypertensive women aged 20 to 34 years, who were married at the time of the survey. Among 267,306 married women aged 20 to 34 years in the NFHS-4, a total of 22,140 were categorized as hypertensive, which constitutes our sample (Fig. 1). The methods were carried out in accordance with the “US Department of Health and Human Services regulations for the protection of human subjects” and relevant national guidelines.

### Measures

The NFHS-4 reports respondents’ average systolic blood pressure (SBP) and diastolic blood pressure (DBP) measures. Blood pressure was measured three times during a single visit with at least 5 min interval between each reading. Respondents were also asked if they were taking any antihypertensive medication to lower their blood pressure. A respondent was categorized as hypertensive if average SBP ≥ 140 mmHg or the average DBP ≥ 90 mmHg or the respondent reported taking antihypertensive medication at the time of the survey. An individual was determined to have untreated hypertension if the average blood pressure measure exceeded the normal threshold, and the individual was not taking antihypertensive medication at the time of the survey.

The NFHS-4 also reports respondents’ age at first marriage. Women who were married before the age of 18 years were identified as child brides. Age at first marriage information was only available for those who were currently married, and was not available for those who were widowed, divorced, or separated at the time of the survey.

### Statistical analysis

We estimated univariate and multivariable logistic regressions to obtain odds ratios (ORs) and adjusted ORs (AORs) in favor of not receiving hypertension treatment. Our dependent variable is a binary variable indicating if the respondent received hypertension treatment or not. The key explanatory variable is another binary variable indicating whether the respondent was married as child (i.e., before age 18 years) or as adult (i.e., at or after age 18 years).

In multivariable logistic model, we controlled for various sociodemographic correlates including age in 3-years interval: 20 to 22 (reference category), 23 to 25, 26 to 28, 29 to 31, and 32 to 34; education attainment: no education (reference category), primary, secondary, and higher; relationship to household head: head (reference category), wife, daughter, daughter-in-law, and other; parity or number of children born: 0 (reference category), 1 to 2, 3 to 4, and 5 + ; current pregnancy status; current breastfeeding (lactation) status; household size: 3 or less (reference category), 4 to 5, 6 to 8, and 9 + ; household wealth index quintiles: poorest (reference category), poorer, middle, richer, and richest; religion: Hindu (reference category), Muslim, Christian, Sikh, Buddhist, and other; caste: not socially or economically backward class (reference category), scheduled caste, scheduled tribe, other backward class; and residence: rural (reference category) and urban. To account for state level differences in health policy and health care access, we also controlled for state fixed effects.

We first estimated the crude ORs and AORs in favor of having untreated hypertension for each of these sociodemographic characteristics in subgroups of women who were married as adults and who were married as children. We then performed Chow test to examine whether the crude ORs and AORs for respective sociodemographic characteristics differ between the two groups. Estimates were obtained using complex survey weights and the level of significance was set to 0.05.

Next, to assess the relationship between child marriage and untreated hypertension, we estimated a univariate specification (model 1) only including the child marriage indicator and a constant. Subsequently we estimated four multivariable specifications as follows: model 2 includes individual level correlates (i.e., age, educational attainment, relationship to household head, parity, current pregnancy status, and current lactation status); model 3 includes household level correlates (i.e., household size, household wealth index quintiles, religion, caste, and residence); model 4 includes both individual and household level correlates; and model 5 includes state fixed effects in addition to individual and household level correlates.

Next, we offered two robustness checks of our analyses. First, instead of the binary child marriage indicator, we used the length of marriage as the key explanatory variable. Since the length of marriage, especially in the context of child marriage, varies broadly across age groups, we standardized the length of marriage using the following formula:

$SML_{i,a\,} = \,\frac{{ML_{i,a} - \overline{{ML_{a} }} }}{{STDV_{a} }}$, where, *SML*_*i,a*_ is the standardized length of marriage for individual *i* of age group *a*, *ML*_*i,a*_ is the actual length of marriage of individual *i* of age group *a*, $\overline{{ML_{a} }}$ is the average length of marriage in age group *a*, and *STDV*_*a*_ is the standard deviation of length of marriage in age group *a*. We estimated models 1 to 5 to assess how one standard deviation increase in length of marriage is associated with the likelihood of having untreated hypertension in our sample.

Second, exploiting the hierarchical nature of NFHS-4 data, we performed a multilevel analysis to account for potential bias in standard errors emanating from clustering of data. Following Jain et al. [15], we estimated a multilevel logistic regression model where individual (level 1) is nested within community (level 2)—defined by Census Enumeration Blocks in urban areas and villages in rural areas, district (level 3), and state (level 4). We thus fitted a four-level random intercept model. Since we are primarily interested in examining the relationship between child marriage and untreated hypertension, we did not examine whether or how community level variables impact the individual level outcome (i.e., untreated hypertension) nor we explored the extent of relative contribution of different levels.

## Results

Among married women aged 20 to 34 years in the NFHS-4, around 8% were hypertensive and 27% of the hypertensive women received treatment at the time of the survey. Around 40% women in this group were married as children. Prevalence of hypertension was 1.5 percentage points higher (*P* < 0.001) among child brides than their peers who were married as adults. Among hypertensive women in the sample, child brides were more likely not to receive treatment compared to women who were married as adults (Fig. 1).

Background characteristics of the study participants are presented in Table 1. Child marriage was more prevalent in older cohorts, in rural areas, among scheduled castes and tribes, and at lower levels of educational attainment and household wealth. Prevalence of untreated hypertension was higher among child brides across most sociodemographic characteristics. The prevalence was significantly higher in older cohorts (age 29–34 years), among women at poorer, middle, and richer households, and among women residing in rural areas.

**Table 1:** Background characteristics of study participants and untreated hypertension prevalence

Characteristic	Share of women				Untreated hypertension prevalence			
	All (*n* = 22,140)	Married as adult (*n* = 12,746)	Married as child (*n* = 9,394)	*P*-value	All (*n* = 22,140)	Married as adult (*n* = 12,746)	Married as child (*n* = 9,394)	*P*-value
Individual								
Age (yr)								
20–22	9.23 (8.68–9.78)	9.61 (8.88–10.35)	8.77 (7.99–9.55)	0.115	65.00 (62.06–67.94)	62.46 (58.54–66.38)	68.37 (63.96–72.77)	0.054
23–25	16.20 (15.47, 16.94)	17.86 (16.84–18.88)	14.20^a)^ (13.12–15.28)	< 0.001	68.06 (65.56–70.55)	67.24 (64.24–70.23)	69.31 (65.05–73.57)	0.434
26–28	20.83 (20.04–21.62)	21.88 (20.74–23.02)	19.56^a)^ (18.40–20.72)	0.006	72.25 (70.24–74.25)	71.26 (68.57–73.95)	73.59 (70.64–76.53)	0.250
29–31	25.74 (24.88–26.60)	23.66 (22.55–24.77)	28.24^a)^ (26.90–29.59)	< 0.001	74.59 (72.80–76.39)	71.06 (68.67–73.45)	78.17^b)^ (75.50–80.84)	< 0.001
32–34	28.00 (27.12–28.88)	26.98 (25.78–28.19)	29.22^a)^ (27.90–30.55)	0.015	76.06 (74.45–77.67)	74.93 (72.75–77.12)	77.31^b)^ (74.99–79.63)	0.131
Education								
No education	26.10 (25.29–26.90)	17.73 (16.81–18.64)	36.21^a)^ (34.95–37.46)	< 0.001	76.76 (75.23–78.29)	76.26 (73.87–78.64)	77.06 (75.22–78.89)	0.580
Primary	15.02 (14.33–15.71)	10.75 (9.97–11.53)	20.17^a)^ (18.98–21.36)	< 0.001	74.49 (72.27–76.70)	73.65 (70.27–77.03)	75.03 (72.13–77.94)	0.538
Secondary	47.83 (46.82–48.84)	53.40 (52.08–54.73)	41.09^a)^ (39.68–42.51)	< 0.001	71.07 (69.67–72.46)	69.26 (67.54–70.98)	73.90^b)^ (71.61–76.19)	0.001
Higher	11.06 (10.34–11.78)	18.12 (17.01–19.24)	2.53^a)^ (1.80–3.26)	< 0.001	66.59 (63.13–70.05)	67.43 (64.19–70.66)	59.33 (43.29–75.37)	0.336
Relation to household head								
Head	3.63 (3.31–3.95)	2.54 (2.19–2.90)	4.94^a)^ (4.40–5.48)	< 0.001	66.92 (62.50–71.35)	61.13 (54.21–68.06)	70.52^b)^ (65.16–75.89)	0.033
Wife	56.81 (55.85–57.77)	49.33 (48.04–50.63)	65.84^a)^ (64.54–67.14)	< 0.001	74.87 (73.60–76.14)	72.95 (71.26–74.63)	76.62^b)^ (74.85–78.38)	0.002
Daughter	5.97 (5.51–6.44)	7.42 (6.71–8.14)	4.22^a)^ (3.65–4.79)	< 0.001	64.74 (61.05–68.44)	64.60 (60.11–69.09)	65.04 (58.45–71.63)	0.913
Daughter-in-law	29.55 (28.67–30.44)	35.96 (34.69–37.24)	21.82^a)^ (20.71–22.92)	< 0.001	70.79 (69.14–72.44)	69.57 (67.56–71.59)	73.22^b)^ (70.48–75.96)	0.033
Other	4.03 (3.66–4.40)	4.73 (4.20–5.26)	3.19^a)^ (2.67–3.70)	< 0.001	69.85 (65.65–74.05)	69.29 (64.31–74.27)	70.85 (63.62–78.08)	0.717
Parity								
0	10.11 (9.52–10.70)	15.27 (14.34–16.21)	3.88^a)^ (3.28–4.48)	< 0.001	73.37 (70.67–76.06)	71.57 (68.58–74.55)	81.92^b)^ (76.11–87.74)	0.003
1–2	58.98 (58.01–59.95)	66.64 (65.43–67.84)	49.73^a)^ (48.22–51.23)	< 0.001	71.55 (70.25–72.85)	69.86 (68.28–71.43)	74.29^b)^ (72.20–76.38)	0.001
3–4	26.63 (25.76–27.49)	16.53 (15.62–17.45)	38.81^a)^ (37.36–40.26)	< 0.001	73.97 (72.20–75.75)	72.61 (69.81–75.42)	74.67 (72.47–76.87)	0.246
5 +	4.29 (3.95–4.63)	1.56 (1.28–1.83)	7.58^a)^ (6.95–8.22)	< 0.001	76.02 (72.55–79.49)	74.02 (64.84–83.19)	76.52 (72.94–80.10)	0.619
Pregnant								
No	94.70 (94.29–95.11)	92.90 (92.27–93.54)	96.87 (96.42–97.32)	< 0.001	73.52 (72.52–74.52)	71.71 (70.44–72.99)	75.61^b)^ (74.18–77,05)	< 0.001
Yes	5.30 (4.89–5.71)	7.10 (6.46–7.73)	3.13 (2.68–3.58)	< 0.001	55.61 (51.62–59.61)	56.57 (51.87–61.27)	52.99 (45.67–60.31)	0.408
Lactating								
No	72.02 (71.17–72.87)	67.53 (66.33–68.73)	77.43^a)^ (76.29–78.58)	< 0.001	73.58 (72.43–74.73)	71.65 (70.16–73.14)	75.62^b)^ (73.95–77.28)	< 0.001
Yes	27.98 (27.13–28.83)	32.47 (31.27–33.67)	22.57^a)^ (21.42–23.71)	< 0.001	69.97 (68.30–71.63)	68.53 (66.45–70.62)	72.46^b)^ (69.94–74.97)	0.016
Household characteristics								
Household size								
3 or less	13.72 (13.00–14.44)	15.82 (14.80–16.83)	11.18^a)^ (10.18–12.18)	< 0.001	74.26 (71.79–76.73)	71.82 (68.69–74.95)	78.43^b)^ (74.31–82.56)	0.013
4–5	44.23 (43.25–45.22)	41.54 (40.19–42.89)	47.49^a)^ (46.05–48.93)	< 0.001	73.19 (71.79–74.59)	71.01 (69.14–72.88)	75.49^b)^ (73.52–77.45)	0.001
6–8	28.59 (27.74–29.44)	28.36 (27.16–29.56)	28.87 (27.70–30.04)	0.556	72.22 (70.59–73.86)	70.43 (68.13–72.73)	74.35^b)^ (72.21–76.50)	0.012
9 +	13.46 (12.78–14.14)	14.28 (13.42–15.15)	12.46^a)^ (11.43–13.49)	0.008	69.56 (66.90–72.22)	68.67 (65.51–71.84)	70.79 (66.26–75.33)	0.449
Wealth index quintiles								
Poorest	18.34 (17.68–19.00)	12.94 (12.22–13.66)	24.86^a)^ (23.79–25.93)	< 0.001	76.06 (74.26–77.85)	74.88 (72.11–77.64)	76.80 (74.61–78.98)	0.264
Poorer	19.49 (18.76–20.22)	16.10 (15.24–16.95)	23.59^a)^ (22.43–24.75)	< 0.001	72.85 (70.95–74.75)	71.17 (68.51–73.83)	74.23^b)^ (71.70–76.75)	0.086
Middle	19.91 (19.13–20.69)	18.94 (17.96–19.91)	21.08^a)^ (19.91–22.25)	0.005	71.68 (69.71–73.64)	69.46 (66.85–72.07)	74.08^b)^ (71.22–76.94)	0.019
Richer	22.68 (21.79–23.58)	25.22 (23.99–26.45)	19.62^a)^ (18.38–20.86)	< 0.001	72.25 (69.98–74.52)	69.98 (67.29–72.66)	75.77^b)^ (71.94–79.61)	0.010
Richest	19.58 (18.70–20.46)	26.81 (25.53–28.09)	10.85^a)^ (9.81–11.89)	< 0.001	70.31 (67.85–72.78)	69.73 (67.03–72.42)	72.07 (66.61–77.52)	0.445
Religion								
Hindu	77.44 (76.52–78.36)	76.73 (75.56–77.89)	78.30 (77.03–79.56)	0.063	72.13 (71.00–73.27)	70.16 (68.73–71.59)	74.47^b)^ (72.83–76.12)	< 0.001
Muslim	16.07 (15.27–16.88)	15.40 (14.43–16.38)	16.88^a)^ (15.75–18.02)	0.044	73.77 (71.68–75.86)	71.82 (68.92–74.73)	75.91 (72.97–78.85)	0.052
Christian	2.44 (2.09–2.78)	2.78 (2.37–3.20)	2.01^a)^ (1.54–2.49)	0.016	69.28 (63.52–75.05)	63.97 (56.68–71.26)	78.14^b)^ (69.26–87.03)	0.006
Sikh	2.21 (2.00–2.41)	3.15 (2.86–3.43)	1.07^a)^ (0.86–1.28)	< 0.001	80.44 (76.91–83.97)	80.81 (76.80–84.82)	79.14 (71.50–86.79)	0.712
Buddhist	0.87 (0.66–1.08)	0.83 (0.57–1.08)	0.92 (0.58–1.27)	0.656	65.68 (52.62–78.75)	61.86 (44.69–79.03)	69.82 (49.92–89.72)	0.554
Other	0.97 (0.64–1.31)	1.11 (0.60–1.63)	0.81 (0.49–1.13)	0.295	84.03 (75.86–92.20)	81.81 (69.90–93.72)	87.72 (77.52–97.93)	0.397
Caste								
None	28.20 (27.28–29.13)	30.07 (28.87–31.28)	25.94^a)^ (24.60–27.28)	< 0.001	73.66 (71.85–75.47)	71.91 (69.47–74.35)	76.11^b)^ (73.39–78.82)	0.024
Scheduled caste	19.77 (18.88–20.66)	18.26 (17.22–19.30)	21.60^a)^ (20.27–22.93)	< 0.001	70.84 (68.51–73.16)	69.18 (66.34–72.03)	72.52 (69.07–75.98)	0.118
Scheduled tribe	9.95 (9.44–10.45)	8.87 (8.24–9.49)	11.25^a)^ (10.52–11.99)	< 0.001	79.34 (77.40–81.29)	78.72 (76.10–81.33)	79.94 (77.18–82.71)	0.510
Other backward class	42.08 (41.06–43.09)	42.80 (41.47–44.13)	41.21 (39.83–42.58)	0.106	71.06 (69.61–72.50)	68.69 (66.76–70.63)	74.02^b)^ (72.01–76.03)	< 0.001
Residence								
Rural	32.82 (32.04–33.60)	60.79 (59.86–61.72)	74.90^a)^ (73.82–75.98)	< 0.001	71.94 (69.83–74.05)	70.21 (68.85–71.58)	75.49^b)^ (74.12–76.86)	0.407
Urban	67.18 (66.40–67.96)	39.21 (38.28–40.14)	25.10^a)^ (24.02–26.18)	< 0.001	72.88 (71.85–73.90)	71.30 (68.94–73.66)	73.15 (69.26–77.04)	< 0.001

95% Confidence intervals are presented in parentheses; estimates were obtained using complex survey weights; shares add to 100 across rows for each characteristic (e.g., age, education, etc.)

^a)^Significantly different (*P* < 0.05) share across the two groups (married as adult vs. married as child

^b)^Statistically different (*P* < 0.05) untreated hypertension prevalence across the two groups (married as adult vs. married as child)

Table 2 presents the crude ORs and AORs in favor of having untreated hypertension for the sociodemographic correlates among women who were married as adults and women who were married as children. In both groups, higher age was found as a risk factor for untreated hypertension. Higher level of education, on the other hand, was found inversely associated with untreated hypertension in both groups. The odds of having untreated hypertension were significantly lower among women who were currently (at the time of survey) pregnant or lactating in both groups. Results of the Chow test suggested that the odds in favor of having untreated hypertension for these factors were not statistically different across the two groups.

**Table 2:** Risk factors of untreated hypertension among women married as adults and as children

Variable	Crude odds ratio (95% confidence interval)			Adjusted odds ratio (95% confidence interval)		
	Married as adult (*n* = 12,746)	Married as child (*n* = 9,394)	*P*-value	Married as adult (*n* = 12,746)	Married as child (*n* = 9,394)	*P*-value
Age (yr)						
20–22	Reference	Reference		Reference	Reference	
23–25	1.234 (0.998–1.525)	1.045 (0.791–1.380)	0.357	1.261^*^ (1.014–1.568)	1.095 (0.843–1.421)	0.512
26–28	1.490^***^ (1.205–1.843)	1.289^*^ (1.008–1.649)	0.381	1.536^***^ (1.218–1.938)	1.290 (0.978–1.701)	0.471
29–31	1.476^***^ (1.206–1.807)	1.657^***^ (1.281–2.142)	0.492	1.497^***^ (1.184–1.893)	1.664^***^ (1.248–2.219)	0.392
32–34	1.797^***^ (1.465–2.204)	1.577^***^ (1.239–2.006)	0.410	1.793^***^ (1.396–2.304)	1.566^**^ (1.155–2.124)	0.527
Education						
No education	Reference	Reference		Reference	Reference	
Primary	0.87 (0.699–1.083)	0.895 (0.745–1.074)	0.844	0.888 (0.712–1.109)	0.904 (0.751–1.089)	0.912
Secondary	0.701^***^ (0.601–0.818)	0.843^*^ (0.722–0.985)	0.981	0.733^***^ (0.612–0.878)	0.877 (0.736–1.047)	0.155
Higher	0.645^***^ (0.532–0.782)	0.434^*^ (0.222–0.850)	0.267	0.658^***^ (0.519–0.833)	0.471^*^ (0.259–0.854)	0.286
Relation to household head						
Head	Reference	Reference		Reference	Reference	
Wife	1.714^***^ (1.265–2.323)	1.370^*^ (1.039–1.805)	0.264	1.857^***^ (1.360–2.535)	1.486^**^ (1.117–1.977)	0.291
Daughter	1.16 (0.817–1.647)	0.778 (0.528–1.144)	0.126	1.494^*^ (1.027–2.172)	1.052 (0.673–1.644)	0.189
Daughter-in-law	1.454^*^ (1.070–1.974)	1.143 (0.855–1.528)	0.247	1.800^***^ (1.279–2.534)	1.516^*^ (1.052–2.184)	0.470
Other	1.434 (0.994–2.070)	1.016 (0.660–1.564)	0.218	1.781^**^ (1.186–2.675)	1.329 (0.789–2.239)	0.319
Parity						
0	Reference	Reference		Reference	Reference	
1–2	0.921 (0.780–1.087)	0.637^*^ (0.424–0.958)	0.098	0.715^***^ (0.586–0.872)	0.572^*^ (0.364–0.899)	0.341
3–4	1.053 (0.863–1.286)	0.651^*^ (0.434–0.974)	0.036	0.665^**^ (0.513–0.863)	0.517^**^ (0.323–0.828)	0.351
5 +	1.132 (0.688–1.861)	0.719 (0.468–1.106)	0.172	0.627 (0.374–1.050)	0.535^*^ (0.313–0.914)	0.767
Pregnant						
No	Reference	Reference		Reference	Reference	
Yes	0.514^***^ (0.421–0.628)	0.364^***^	0.058	0.508^**^ (0.411–0.627)	0.388^**^ (0.279–0.539)	0.173
Lactating						
No	Reference	Reference		Reference	Reference	
Yes	0.862^*^ (0.766–0.970)	0.848^*^ (0.728–0.989)	0.870	0.925 (0.801–1.068)	1.016 (0.849–1.215)	0.421
Household size						
3 or less	Reference	Reference		Reference	Reference	
4–5	0.961 (0.802–1.151)	0.847 (0.659–1.087)	0.429	0.906 (0.749–1.096)	0.806 (0.626–1.038)	0.415
6–8	0.934 (0.772–1.130)	0.797 (0.612–1.039)	0.343	0.954 (0.760–1.198)	0.775 (0.572–1.049)	0.250
9 +	0.860 (0.695–1.064)	0.666^*^ (0.480–0.925)	0.197	0.899 (0.696–1.162)	0.670 (0.427–1.053)	0.223
Wealth index quintiles						
Poorest	Reference	Reference		Reference	Reference	
Poorer	0.828 (0.680–1.008)	0.87 (0.726–1.043)	0.710	0.880 (0.714–1.084)	0.876 (0.724–1.059)	0.871
Middle	0.763^**^ (0.630–0.925)	0.864 (0.710–1.050)	0.363	0.820 (0.661–1.017)	0.888 (0.714–1.105)	0.626
Richer	0.782^*^ (0.644–0.949)	0.945 (0.743–1.202)	0.212	0.819 (0.648–1.036)	1.015 (0.793–1.298)	0.280
Richest	0.773^**^ (0.636–0.938)	0.78 (0.579–1.049)	0.960	0.766^*^ (0.593–0.991)	0.876 (0.647–1.187)	0.585
Religion						
Hindu	Reference	Reference		Reference	Reference	
Muslim	1.084 (0.926–1.270)	1.08 (0.899–1.297)	0.976	1.072 (0.900–1.276)	1.134 (0.922–1.394)	0.724
Christian	0.755 (0.547–1.043)	1.225 (0.724–2.074)	0.145	0.688^*^ (0.487–0.974)	1.119 (0.641–1.952)	0.117
Sikh	1.791^***^ (1.372–2.339)	1.301 (0.812–2.084)	0.255	1.918^***^ (1.434–2.566)	1.305 (0.791–2.155)	0.166
Buddhist	0.69 (0.332–1.433)	0.793 (0.307–2.046)	0.820	0.651 (0.300–1.411)	0.889 (0.338–2.338)	0.646
Other	1.913 (0.857–4.269)	2.449 (0.947–6.337)	0.703	1.673 (0.734–3.813)	2.192 (0.797–6.029)	0.732
Caste						
None	Reference	Reference		Reference	Reference	
Scheduled caste	0.877 (0.733–1.049)	0.829 (0.661–1.039)	0.695	0.889 (0.732–1.081)	0.831 (0.646–1.068)	0.729
Scheduled tribe	1.445^***^ (1.188–1.756)	1.251 (0.995–1.574)	0.340	1.465^***^ (1.174–1.829)	1.199 (0.928–1.550)	0.214
Other backward class	0.857^*^ (0.737–0.997)	0.894 (0.746–1.072)	0.721	0.881 (0.754–1.030)	0.919 (0.761–1.111)	0.848
Residence						
Rural	Reference	Reference		Reference	Reference	
Urban	1.054 (0.923–1.203)	0.885 (0.716–1.093)	0.153	1.157 (0.991–1.352)	0.864 (0.701–1.064)	0.031

Estimates were obtained using complex survey weights; the P-values are indicative of whether the odds ratios across the two groups are statistically different

^*^P < 0.05

^**^P < 0.01

^***^P < 0.001

The ORs and AORs in favor of untreated hypertension from the logistic regressions are presented in Table 3. Child brides at young adulthood were 1.24 times more likely to have untreated hypertension than their peers who were married as adults. The AORs became slightly smaller (range, 1.23–1.12) when individual and household level correlates and state fixed effects were controlled for. Among individual correlates, older age was a significant predictor of untreated hypertension. Compared to being the head of the household, women in other roles (e.g., wife or daughter-in-law) were at greater risk of not receiving treatment for hypertensive condition. Higher educational attainment, on the other hand, was associated with lower risk of untreated hypertension. Current pregnancy status, on the other hand, was found associated with lower risk of untreated hypertension. At household level, the risk of not receiving treatment was relatively lower at wealthier households.

**Table 3:** Odds ratios and adjusted odds ratios in favor of untreated hypertension from logistic regression (*n* = 22,140)

Variable	Model 1	Model 2	Model 3	Model 4	Model 5
Child marriage	1.241^***^ (1.131–1.361)	1.148^*^ (1.032–1.277)	1.224^***^ (1.109–1.351)	1.160^**^ (1.042–1.290)	1.123^*^ (1.007–1.251)
Age (yr)	-				
20–22		Reference		Reference	Reference
23–25		1.183^*^ (1.000–1.400)		1.189^*^ (1.006–1.406)	1.197^*^ (1.011–1.418)
26–28		1.427^***^ (1.198–1.699)		1.445^***^ (1.210–1.725)	1.423^***^ (1.188–1.704)
29–31		1.560^***^ (1.307–1.862)		1.598^***^ (1.334–1.914)	1.573^***^ (1.312–1.887)
32–34		1.685^***^ (1.396–2.033)		1.718^***^ (1.414–2.087)	1.657^***^ (1.361–2.018)
Education	-				
No education		Reference		Reference	Reference
Primary		0.872 (0.754–1.008)		0.899 (0.776–1.040)	0.843^*^ (0.726–0.977)
Secondary		0.753^***^ (0.670–0.847)		0.802^***^ (0.707–0.910)	0.785^***^ (0.690–0.892)
Higher		0.609^***^ (0.501–0.741)		0.664^***^ (0.541–0.815)	0.665^***^ (0.543–0.814)
Relation to household head	-				
Head		Reference		Reference	Reference
Wife		1.536^***^ (1.244–1.897)		1.605^***^ (1.296–1.989)	1.428^***^ (1.143–1.784)
Daughter		1.124 (0.864–1.462)		1.234 (0.931–1.636)	1.128 (0.842–1.512)
Daughter-in-law		1.440^**^ (1.158–1.791)		1.583^***^ (1.236–2.028)	1.365^*^ (1.056–1.765)
Other		1.354^*^ (1.018–1.802)		1.503^*^ (1.089–2.074)	1.303 (0.933–1.821)
Parity	-				
0		Reference		Reference	Reference
1–2		0.660^***^ (0.554–0.787)		0.675^***^ (0.565–0.808)	0.705^***^ (0.590–0.843)
3–4		0.588^***^ (0.479–0.722)		0.613^***^ (0.497–0.758)	0.641^****^ (0.518–0.792)
5 +		0.586^***^ (0.443–0.775)		0.599^***^ (0.446–0.804)	0.633^**^ (0.471–0.852)
Pregnant	-				
No		Reference		Reference	Reference
Yes		0.480^***^ (0.402–0.574)		0.469^***^ (0.392–0.562)	0.480^***^ (0.399–0.577)
Lactating	-				
No		Reference		Reference	Reference
Yes		0.984 (0.877–1.103)		0.968 (0.863–1.086)	0.947 (0.843–1.065)
Household size	-				
3 or less			Reference	Reference	Reference
4–5			0.927 (0.804–1.070)	0.875 (0.755–1.015)	0.876 (0.754–1.018)
6–8			0.882 (0.757–1.029)	0.891 (0.744–1.066)	0.882 (0.736–1.058)
9 +			0.798^*^ (0.663–0.961)	0.814 (0.644–1.030)	0.779^*^ (0.614–0.990)
Wealth index quintiles	-				
Poorest			Reference	Reference	Reference
Poorer			0.875 (0.762–1.005)	0.878 (0.761–1.014)	0.877 (0.757–1.016)
Middle			0.847^*^ (0.734–0.977)	0.848^*^ (0.726–0.990)	0.911 (0.775–1.070)
Richer			0.873 (0.748–1.019)	0.890 (0.750–1.057)	0.963 (0.802–1.157)
Richest			0.786^**^ (0.656–0.943)	0.817^*^ (0.672–0.992)	0.916 (0.739–1.136)
Religion	-				
Hindu			Reference	Reference	Reference
Muslim			1.074 (0.944–1.222)	1.098 (0.960–1.255)	1.027 (0.890–1.186)
Christian			0.803 (0.607–1.062)	0.829 (0.617–1.114)	0.927 (0.648–1.326)
Sikh			1.850^***^ (1.449–2.363)	1.744^***^ (1.361–2.234)	1.139 (0.810–1.604)
Buddhist			0.758 (0.422–1.360)	0.741 (0.407–1.349)	0.689 (0.367–1.293)
Other			1.794 (0.957–3.365)	1.838 (0.969–3.485)	1.570 (0.831–2.967)
Caste	-				
None			Reference	Reference	Reference
Scheduled caste			0.858 (0.733–1.004)	0.859 (0.733–1.007)	0.953 (0.813–1.118)
Scheduled tribe			1.346^***^ (1.137–1.594)	1.315^**^ (1.109–1.558)	1.360^***^ (1.138–1.626)
Other backward class			0.892 (0.792–1.005)	0.891 (0.791–1.005)	1.032 (0.911–1.169)
Residence	-				
Rural			Reference	Reference	Reference
Urban			1.062 (0.932–1.211)	1.030 (0.903–1.175)	1.053 (0.919–1.206)
State Fixed Effect	No	No	No	No	Yes

Values are presented as odds ratio or adjusted odds ratio (95% confidence interval); estimates were obtained using complex survey weights

^*^P < 0.05

^**^P < 0.01

^***^P < 0.001

Table 4 reports the relationship between untreated hypertension and the standardized length of marriage. A standard deviation increase in length of marriage was associated with 1.12 times increase in odds of having untreated hypertension. The relationship persisted when individual and household level correlates as well as state fixed effects were accounted for in the model. In each age group, the length of marriage was significantly higher (*P* < 0.001) for those who were married as children compared to those who were married as adults. Thus, the continuous “length of marriage” deemed as a good proxy for dichotomous child marriage indicator. Our results were robust to this continuous specification, reinforcing the relationship between child marriage and untreated hypertension.

**Table 4:** Odds ratios and adjusted odds ratios in favor of untreated hypertension for length of marriage and other covariates (*n* = 22,140)

Variable	Model 1	Model 2	Model 3	Model 4	Model 5
Length of marriage	1.122^***^ (1.073–1.174)	1.108^***^ (1.044–1.177)	1.116^***^ (1.061–1.173)	1.117^***^ (1.051–1.187)	1.099^**^ (1.034–1.168)
Age (yr)	-		-		
20–22		Reference		Reference	Reference
23–25		1.198^*^ (1.012–1.418)		1.204^*^ (1.018–1.425)	1.211^*^ (1.023–1.435)
26–28		1.471^***^ (1.233–1.756)		1.493^***^ (1.248–1.786)	1.465^***^ (1.221–1.758)
29–31		1.645^***^ (1.373–1.972)		1.692^***^ (1.406–2.035)	1.653^***^ (1.372–1.992)
32–34		1.783^***^ (1.470–2.163)		1.825^***^ (1.495–2.228)	1.748^***^ (1.428–2.141)
Education	-		-		
No education		Reference		Reference	Reference
Primary		0.876 (0.758–1.013)		0.902 (0.779–1.045)	0.845^*^ (0.728–0.981)
Secondary		0.764^***^ (0.680–0.858)		0.813^**^ (0.716–0.922)	0.7*** (0.699–0.902)
Higher		0.636^***^ (0.520–0.779)		0.69*** (0.563–0.856)	0.69*** (0.562–0.850)
Relation to household head	-		-		
Head		Reference		Reference	Reference
Wife		1.528^***^ (1.237–1.888)		1.59*** (1.289–1.979)	1.421^**^ (1.137–1.775)
Daughter		1.136 (0.873–1.477)		1.253 (0.945–1.661)	1.143 (0.852–1.533)
Daughter-in-law		1.449^***^ (1.165–1.802)		1.599^***^ (1.247–2.049)	1.377^*^ (1.064–1.782)
Other		1.364^*^ (1.025–1.816)		1.520^*^ (1.101–2.100)	1.316 (0.942–1.839)
Parity	-		-		
0		Reference		Reference	Reference
1–2		0.617^***^ (0.512–0.744)		0.629^***^ (0.522–0.759)	0.662^***^ (0.548–0.799)
3–4		0.538^***^ (0.430–0.674)		0.559^***^ (0.445–0.701)	0.58^***^ (0.468–0.740)
5 +		0.530^***^ (0.393–0.713)		0.539^***^ (0.396–0.732)	0.57^***^ (0.421–0.784)
Pregnant	-		-		
No		Reference		Reference	Reference
Yes		0.489^***^ (0.409–0.585)		0.47*** (0.399–0.573)	0.488^***^ (0.406–0.587)
Lactating	-		-		
No		Reference		Reference	Reference
Yes		1.017 (0.905–1.142)		1.003 (0.892–1.129)	0.979 (0.868–1.104)
Household size	-	-			
3 or less			Reference	Reference	Reference
4–5			0.918 (0.794–1.061)	0.871 (0.751–1.011)	0.873 (0.751–1.015)
6–8			0.875 (0.749–1.023)	0.886 (0.739–1.062)	0.879 (0.732–1.055)
9 +			0.791^*^ (0.656–0.954)	0.808 (0.638–1.022)	0.775^*^ (0.610–0.985)
Wealth index quintiles	-	-			
Poorest			Reference	Reference	Reference
Poorer			0.882 (0.768–1.013)	0.882 (0.765–1.018)	0.880 (0.759–1.019)
Middle			0.853^*^ (0.739–0.985)	0.850^*^ (0.727–0.993)	0.912 (0.776–1.072)
Richer			0.882 (0.756–1.029)	0.892 (0.752–1.059)	0.966 (0.804–1.160)
Richest			0.801^*^ (0.667–0.962)	0.818^*^ (0.673–0.994)	0.917 (0.740–1.137)
Religion	-	-			
Hindu			Reference	Reference	Reference
Muslim			1.068 (0.938–1.215)	1.100 (0.962–1.258)	1.028 (0.891–1.187)
Christian			0.816 (0.615–1.082)	0.841 (0.625–1.132)	0.939 (0.655–1.346)
Sikh			1.847^**^ (1.446–2.360)	1.763^**^ (1.375–2.260)	1.143 (0.813–1.609)
Buddhist			0.748 (0.417–1.341)	0.733 (0.404–1.329)	0.684 (0.366–1.278)
Other			1.789 (0.957–3.344)	1.835 (0.970–3.471)	1.570 (0.833–2.957)
Caste	-	-			
None			Reference	Reference	Reference
Scheduled caste			0.853^*^ (0.729–0.999)	0.859 (0.733–1.006)	0.953 (0.813–1.117)
Scheduled tribe			1.340^***^ (1.132–1.587)	1.317^**^ (1.111–1.561)	1.361^***^ (1.138–1.627)
Other backward class			0.888 (0.788–1.001)	0.891 (0.790–1.004)	1.032 (0.911–1.169)
Residence	-	-			
Rural			Reference	Reference	Reference
Urban			1.065 (0.934–1.214)	1.036 (0.908–1.182)	1.058 (0.924–1.212)
State Fixed Effect	No	No	No	No	Yes

Values are presented as odds ratio or adjusted odds ratio (95% confidence interval); estimates were obtained using complex survey weights; length of marriage is standardized using mean and standard deviations of respective age groups

^*^P < 0.05

^**^P < 0.01

^***^P < 0.001

Results of the multilevel logistic regressions are presented in Table 5. For child brides, the odds of having untreated hypertension were 1.26 times that of those who were married as adults. The AORs after controlling for individual and household level correlates were very similar. The multilevel analysis revealed that there is substantial community level variation (interclass correlation coefficient ≈34% at the community level) in untreated hypertension prevalence among married young adult women in India. Our original set of results that child brides at young adult age are at greater risk of having untreated hypertension, remained significant even after taking into account the hierarchical nature of data; thus, demonstrating the robustness of our findings.

**Table 5:** Odds ratios and adjusted odds ratios in favor of untreated hypertension from multilevel logistic regression

Variable	Null model	Model 1	Model 2	Model 3	Model 4
Child marriage	-	1.255^***^ (1.141–1.380)	1.153^**^ (1.041–1.277)	1.228^***^ (1.107–1.363)	1.154^**^
Age (yr)	-	-		-	
20–22			Reference		Reference
23–25			1.232^*^ (1.048–1.448)		1.240^*^ (1.048–1.467)
26–28			1.391^***^ (1.177–1.643)		1.407^***^ (1.178–1.680)
29–31			1.590^***^ (1.326–1.905)		1.621^***^ (1.340–1.962)
32–34			1.587^***^ (1.279–1.969)		1.622^***^ (1.290–2.041)
Education	-	-		-	
No education			Reference		Reference
Primary			0.796^***^ (0.703–0.901)		0.824^**^ (0.732–0.928)
Secondary			0.694^***^ (0.612–0.786)		0.734^***^ (0.654–0.824)
Higher			0.634^***^ (0.542–0.741)		0.667^***^ (0.558–0.798)
Relation to household head	-	-		-	
Head			Reference		Reference
Wife			1.257^**^ (1.063–1.488)		1.288^**^ (1.086–1.526)
Daughter			0.869 (0.671–1.127)		0.910 (0.712–1.162)
Daughter-in-law			1.169 (0.997–1.370)		1.239^*^ (1.045–1.468)
Other			0.868 (0.659–1.143)		0.925 (0.712–1.202)
Parity	-	-		-	
0			Reference		Reference
1–2			0.673^***^ (0.570–0.794)		0.686^***^ (0.582–0.808)
3–4			0.615^***^ (0.516–0.734)		0.633^***^ (0.526–0.761)
5 +			0.586^***^ (0.465–0.737)		0.593^***^ (0.460–0.766)
Pregnant	-	-		-	
No			Reference		Reference
Yes			0.470^***^ (0.388–0.571)		0.465^***^ (0.382–0.566)
Lactating	-	-		-	
No			Reference		Reference
Yes			0.956 (0.886–1.032)		0.949 (0.881–1.022)
Household size	-	-	-		
3 or less				Reference	Reference
4–5				0.924 (0.808–1.057)	0.905 (0.784–1.044)
6–8				0.881 (0.759–1.022)	0.932 (0.778–1.118)
9 +				0.797^**^ (0.695–0.914)	0.888 (0.743–1.061)
Wealth index quintiles	-	-	-		
Poorest				Reference	Reference
Poorer				0.843^*^ (0.735–0.968)	0.893 (0.780–1.021)
Middle				0.798^***^ (0.727–0.876)	0.857^**^ (0.779–0.943)
Richer				0.785^***^ (0.682–0.904)	0.867 (0.747–1.006)
Richest				0.766^***^ (0.658–0.890)	0.867 (0.726–1.036)
Religion	-	-	-		
Hindu				Reference	Reference
Muslim				0.933 (0.839–1.038)	0.938 (0.827–1.065)
Christian				0.804 (0.641–1.009)	0.838 (0.669–1.051)
Sikh				0.893 (0.569–1.400)	0.899 (0.578–1.398)
Buddhist				1.122 (0.815–1.546)	1.106 (0.810–1.510)
Other				0.973 (0.664–1.426)	0.987 (0.679–1.433)
Caste	-	-	-		
None				Reference	Reference
Scheduled caste				0.967 (0.853–1.096)	0.968 (0.853–1.098)
Scheduled tribe				1.395^**^ (1.114–1.746)	1.398^**^ (1.119–1.746)
Other backward class				1.050 (0.937–1.177)	1.047 (0.931–1.178)
Residence	-	-	-		
Rural				Reference	Reference
Urban				1.088 (0.961–1.231)	1.059 (0.936–1.200)
Random effects^a)^	-	-	-		
Variance					
State level	0.252 (0.119–0.384)	0.250 (0.116–0.384)		0.235 (0.100–0.371)	0.226 (0.129–0.396)
District level	0.246 (0.130–0.362)	0.248 (0.132–0.364)		0.242 (0.125–0.360)	0.237 (0.145–0.388)
Community level^b^^)^	1.220 (0.871–1.570)	1.214 (0.859–1.568)		1.210 (0.855–1.565)	1.215 (0.900–1.641)
ICC					
State level	0.050 (0.03–0.082)	0.050 (0.03–0.083)	0.048 (0.029–0.081)	0.047 (0.027–0.081)	0.045 (0.026–0.077)
District level	0.099 (0.073–0.134)	0.100 (0.073–0.134)	0.097 (0.071–0.132)	0.096 (0.07–0.131)	0.093 (0.067–0.128)
PSU level	0.343 (0.286–0.406)	0.342 (0.284–0.406)	0.341 (0.282–0.405)	0.339 (0.28–0.404)	0.338 (0.278, 0.403)

Values are presented as odds ratio or adjusted odds ratio (95% confidence interval); standard errors were adjusted for 36 clusters in state

ICC, interclass correlation coefficient; PSU, primary sampling units

^a^^)^Sample size: state level, *n* = 36; district level, *n* = 640; PSU level, *n* = 14,087; individual level, *n* = 22,140

^b)^Community level refers to PSU, which are Census Enumeration Blocks in urban areas and villages in rural areas

^**^P < 0.05

^**^P < 0.01

^***^P < 0.001

## Discussion

The study results demonstrate the higher likelihood of having untreated hypertension among the young Indian women who were married during their childhood. Although hypertension can easily be diagnosed and treated early [16], this result shows that young women who got married early either are not screened for hypertension or if diagnosed hypertensive they are not treated for it. In traditional Indian society child marriage is pervasive and it has negative consequences on women’s autonomy, economic empowerment, and both maternal and child health [17]. Young brides are often subject to intimate partner violence and has lower status in the family [17]. Lack of women’s autonomy was found to be associated with lesser health seeking behavior among reproductive age women [18]. This lack of autonomy may be a big reason for the young women not to be treated for hypertension. It is further substantiated by the fact that in our study results that women who are head of their households had lower odds of having untreated hypertension.

In our study we have found the protective effect of education from the untreated hypertension. Child marriage also has intergenerational effect on the women and their child in the form of lower attainment of education and poor condition of their health [19]. Young women who are deprived of schooling also has limited opportunities for creating a social network [18], which can be source of information including health related information. This lack of awareness may also contribute to the untreated hypertension among young women.

We also found that the prevalence of untreated hypertension was lower among women who were pregnant and lactating at the time of the survey. This may be because these women might receive treatment for hypertension as part of antenatal care. Even among the pregnant and lactating women, child brides had higher odds of not receiving treatment for hypertension since women who were married as children in India were significantly less likely to receive antenatal care [20]. The higher likelihood of untreated hypertension among women in 30 s, who has a lower fertility rate compared to women in 20 s, and thereby less likely to receive antenatal care, points out another important public health concern. The primary focus of women’s health in many developing countries had been the maternal and child health issues [21], and women’s general health were often neglected. In the era of global epidemic of noncommunicable diseases, expansion of health care services for women beyond the maternal and child health is a priority; and special focus is required to eliminate the additional barriers of healthcare access and utilization among women who were married as children.

Uncontrolled hypertension exacerbates the risk of all-cause and cardiovascular disease mortality [22]. Population level management of hypertension, therefore, is extremely important to attain the United Nations Sustainable Development Goals target of reducing NCD related premature mortality by one-third by 2030 [23]. Uncontrolled hypertension may lead to a wide range of chronic conditions affecting heart, vascular system, and kidney [24]. NCD treatment in LMICs are often associated with catastrophic health expenditure and consumption displacement of essential commodities [25, 26]. The higher risk of financial stress and impoverishment related to NCDs may further deteriorate child brides’ poor socioeconomic conditions.

Although Indian government enacted the Prohibition of Child Marriage Act in 2007, child marriage is still prevalent in India [27]. Despite notable progress achieved in preventing child marriage over the last couple of decades, a significant share of currently living women in India were married as children. Besides stringently implementing the law to prevent child marriage, we need to focus on the huge population of young women who were married as children and suffering from untreated hypertension in India. Hypertension screening can easily be done by the grassroot level health workers and if young women are found hypertensive proper referral and treatment guidelines should be implemented. The public health implications of our study, thus, are twofold: first we illustrated a long-term health disparity associated with child marriage, which adds to the long list of negative consequences of child marriage and demands strengthening efforts to eliminate the practice of child marriage worldwide; and second, our findings identified a vulnerable group who are in need of apt policy attention for hypertension care.

Although the study explores a novel research question on the association between child marriage and hypertension in later life, there are at least three limitations. First, since we are using a cross-sectional data, we cannot establish a causal connection between child marriage and hypertension. Second, the hypertension status was measured by one-time measure of the blood pressure and the respondent’s response to their previous diagnosis, which is not clinically vetted. Third, this study cannot shed light on the exact socio-biological mechanism of developing hypertension in young women. However, further study with longitudinal examination of women who got married in their childhood is warranted to explore the exact pathway of hypertension in this population.

## Conclusions

This study highlights the importance of regular screening for hypertension among young women who were married in their childhood. Untreated hypertension can culminate into cardiovascular morbidity and mortality and thereby cause huge economic and social losses [28]. Morbidity and mortality of young women not only increases the disability-adjusted life years but also creates an intergenerational harmful effect on their children’s health and wellbeing. Therefore, young women who got married in their childhood should be targeted for regular screening and proper referral and treatment to avoid further detrimental effects of elevated blood pressure.

## Data Availability

The dataset used in this study is available from the US Agency for International Development's Demographic and Health Surveys (DHS) Program. The DHS datasets are free to download and use upon registering at the DHS program website: https://dhsprogram.com/data/new-user-registration.cfm

## Abbreviations

AOR: Adjusted Odds Ratio
DBP: Diastolic Blood Pressure
ICC: Interclass Correlation Coefficient
LMIC: Low-and-Middle Income Country
NCDs: Noncommunicable Disease
NFHS-4: National Family Health Survey
OR: Odds Ratio
PSU: Primary Sampling Units
SBP: Systolic Blood Pressure
